# Point Cloud Completion Network Applied to Vehicle Data

**DOI:** 10.3390/s22197346

**Published:** 2022-09-27

**Authors:** Xuehan Ma, Xueyan Li, Junfeng Song

**Affiliations:** 1 State Key Laboratory of Integrated Optoelectronics, College of Electronic Science and Engineering, Jilin University, Changchun 130012, China; 2 Peng Cheng Laboratory, Shenzhen 518000, China

**Keywords:** point clouds, neural networks, transformer

## Abstract

With the development of autonomous driving, augmented reality, and other fields, it is becoming increasingly important for machines to more accurately and comprehensively perceive their surrounding environment. LiDAR is one of the most important tools used by machines to obtain information about the surrounding environment. However, because of occlusion, the point cloud data obtained by LiDAR are not the complete shape of the object, and completing the incomplete point cloud shape is of great significance for further data analysis, such as classification and segmentation. In this study, we examined the completion of a 3D point cloud and improved upon the FoldingNet auto-encoder. Specifically, we used the encoder–decoder architecture to design our point cloud completion network. The encoder part uses the transformer module to enhance point cloud feature extraction, and the decoder part changes the 2D lattice used by the A network into a 3D lattice so that the network can better fit the shape of the 3D point cloud. We conducted experiments on point cloud datasets sampled from the ShapeNet car-category CAD models to verify the effectiveness of the various improvements made to the network.

## 1. Introduction

With improvements in the performance of point cloud data acquisition equipment such as LiDAR, point clouds have become increasingly widely used in the fields of robotic automated driving and virtual reality, among others. It has become one of the most important data formats in 3D representation, and has been widely used in tasks such as object classification [1,2,3,4], segmentation [2,4,5], pose estimation [6,7], object recognition [8], and object detection [9,10].

Point cloud processing technology is also widely used in extended reality fields such as virtual, augmented, and mixed reality. Extended reality technologies represent a paradigm that enhances and supports Industry 4.0 in diverse settings [11,12]. Digital twins are one of the disruptive technologies associated with the Industry 4.0 concept. Combining the advanced point cloud processing algorithms with cameras and sensors [13] will facilitate the development of Industry 4.0 and related applications [14,15].

There are three typical representations of 3D data: voxels [16], meshes [17,18], and point clouds [19]. A voxel-based representation can apply a traditional convolutional neural network (CNN) to 3D data. However, as the resolution increases, the storage and computing resource consumption of the voxel method significantly increases. Therefore, it is not suitable for high-resolution point cloud reconstruction. Compared with a voxel, a point cloud is a simpler and more unified structure; it can represent 3D shapes more efficiently and is easier to manipulate when geometric transformations are performed.

Real-world point cloud data are usually incomplete. For example, owing to occlusion or interference, the point cloud data scanned by LiDAR are partially incomplete, resulting in the loss of geometric information of the objects. The incompleteness of point cloud data affects further processing. Therefore, converting a partial point cloud into a complete point cloud is of great value for downstream applications such as classification, segmentation, and object detection.

The difficulty in processing point clouds is that the point cloud is disordered and rotationally invariant; therefore, it is difficult to apply traditional convolution operations to point clouds. PointNet [2] and PointNet++ [4], proposed by Qi et al., provide solutions to the point cloud disorder problem. They directly operate on the point cloud for classification and segmentation, avoiding the loss of information caused by the point cloud during data format conversion. FoldingNet [20] contains an auto-encoder where the encoder part can extract the global features of the point cloud and the decoder part can recover data of the original point cloud as accurately as possible from the global features. These two studies laid the foundation for a point cloud completion network. Yuan et al. [21] also adopted an encoder–decoder architecture. The difference is that their decoder adopts a two-stage generation framework to generate a detailed point cloud. The aforementioned networks directly output the complete point cloud; however, the unoccluded parts need not be generated by the network. In addition, the decoder of FoldingNet folds 2D lattices into 3D shapes, which are more difficult to learn and train. In recent years, transformers [22] have achieved excellent results in the fields of natural language processing and computer vision [23]. Inspired by this, Zhao et al. [24] applied transformers to point cloud scenes and proposed point transformers. Point transformers have demonstrated excellent performance in tasks such as classification and segmentation. However, the encoder of most completion networks adopts a multilayer perceptron (MLP) or a similar architecture, and the feature extraction ability is limited.

In response to the above problems, we designed several improvements to the existing networks. The main contributions of this study are as follows:

(1) We think that the unoccluded part of the point cloud does not need to be generated by the network; hence, our network only predicts the occluded part and then stitches the output of the network with the unoccluded part into a complete point cloud of the shape.
(2) We replaced the 2D lattice in the FoldingNet decoder with a 3D lattice and directly deformed the three-dimensional point cloud into a point cloud of the occluded part. This can simplify network training and improve network performance.
(3) The feature extraction capability of the MLP encoder is limited, and to improve it, we used a transformer module as the encoder of our completion network.

The Section 2 of this paper introduces the related work of point cloud completion, the Section 3 introduces our network model and loss function in detail, the Section 4 introduces the specific implementation and results of the experiment, the Section 5 discusses possible further improvements for the network, and the Section 6 summarizes the study.

## 2. Related Work

Point cloud completion methods can be divided into two categories: traditional and learning-based point cloud completion methods. Traditional methods include geometry- and template-based methods. Learning-based methods mainly use encoder–decoder architecture networks or multisegment generation networks.

### 2.1. Traditional Completion Methods

Geometry-based methods use information from incomplete input shapes to obtain complete shapes. It needs the geometric properties of the shape, such as the continuity of the surface and the symmetry of the shape. Surface-oriented methods [25,26] employ smooth interpolation to fill incomplete holes on the surfaces of the shape. Symmetry-based methods [7,27] first identify the symmetry axis and recurrent structures of the shape and then copy the shape of the unoccluded part to the missing part. These methods require that the missing parts can be inferred from the unoccluded parts; therefore, they are only suitable for data that are not severely occluded. However, real-world data are often severely occluded, which sometimes makes these methods ineffective. Model-based methods complete shapes by matching incomplete input shapes to models in large databases. The direct retrieval method [28,29] directly matches the input with the model in the database as the final result of the completion. Partial retrieval methods [17,30] divide the input into several parts to match the models in the database and then combine the matching results to generate the final completion result. Deform-based methods [31,32] deform retrieved shapes to obtain shapes that better match the input. The geometric primitive method [33,34] uses geometric primitives instead of large databases and matches the input with geometric primitives to synthesize the final shape.

The advantage of the traditional method is that it is easy to implement with a simple algorithm. The disadvantage is that when the incomplete area of the input point cloud is too large, the geometry of the missing area cannot be estimated.

### 2.2. Learning-Based Methods

Learning-based methods use neural networks and large amounts of data for shape completion. Some studies [35,36] represented shapes as voxels, and generalized traditional 2D convolution to 3D convolution. The PointNet [2] and PointNet++ [4] networks solve the problems caused by the disorder and rotation invariance of point clouds and obtain high-dimensional features of point clouds. The decoder of FoldingNet [20] demonstrated the feasibility of restoring point clouds from high-dimensional features. PCN [21] uses an encoder similar to that of FoldingNet to extract features and employs two stages in the decoder to generate high-density point clouds. TopNet [37] models the point cloud generation process as the growth of a rooted tree, and uses a hierarchical point cloud generation decoder. SA-Net [38] applies a self-attention mechanism to the network, which effectively preserves local information. SoftPoolNet [3] replaces max pooling with SoftPool and retains more information. PF-Net [39] uses an idea similar to fractal geometry, taking an incomplete point cloud as the input, but only outputting the missing part of the point cloud. SnowflakeNet [40] models the generation of a complete point cloud as a snowflake-like growth of points in a 3D space, revealing local geometric details.

The main advantage of the learning-based methods is that they have strong applicability, and there is no restriction on incomplete shapes or incomplete areas in the input point cloud. Even if the incompleteness is serious, it can be completed. The disadvantage is that they require a large amount of data for training. If the training data are too small, the learning-based methods cannot fit the shape well.

## 3. Methods

**Figure 1:**
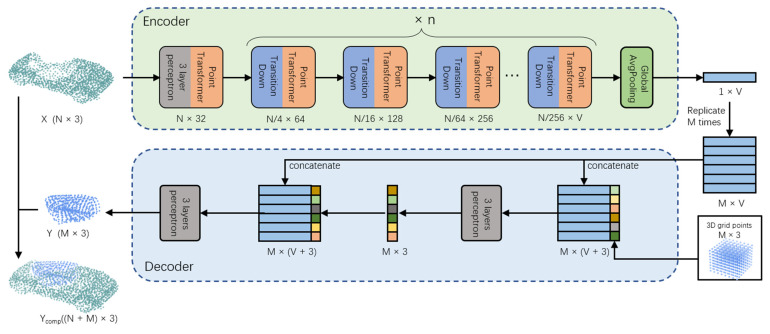
Network architecture mainly consists of a transformer-based encoder and an improved fold-based decoder.

This section introduces the network architecture design. Our network predicts the point cloud of the occluded part from that of the unoccluded input part. Figure 1 illustrates the architecture of the network. The encoder uses the point cloud *X* of the unoccluded part as input and outputs a one-dimensional global feature vector. According to the global vector, the decoder deforms the 3D lattice into the missing part of the point cloud, *Y_occ_*. We optimized the network by calculating the loss between the *Y_occ_* and ground truth (GT), which retains only the occluded part, *GT_occ_*. Finally, the *Y_occ_* was stitched with the unoccluded part to obtain the complete point cloud, *Y_comp_*. We evaluated the completion performance of the network by computing the loss between the *Y_comp_* and GT. Next, we detail the architecture of the encoder, decoder, and use of the loss functions.

### 3.1. Encoder

**Figure 2:**
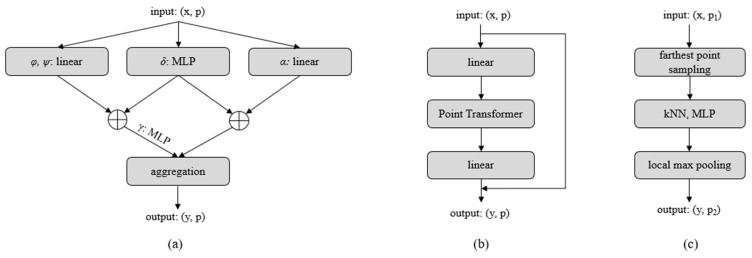
Network architecture of the transformer encoder comprises (**a**) point transformer layer, (**b**) point transformer block, and (**c**) transition down block.

To ensure that the encoder has excellent feature extraction, we used the point transformer [24] proposed by Zhao et al. as the encoder. As shown in Figure 2, the unoccluded part point cloud *X* passes through an MLP-point transformer module and is transformed into an N×32 matrix. It is then processed using the transition down and point transformer modules n times, and an N/256×32 matrix is obtained, where V is 32 × 4^n^. Finally, average pooling is performed on this matrix, and a global feature with the shape (1, V) is obtained.

The specific architecture of the point transformer layer is shown in Figure 2a. The self-attention feature, denoted as $y_{i}$, of features corresponding to each point in the point cloud, denoted as $x_{i}$, is calculated with the feature set $\chi_{i}$ of k-nearest neighbors:

(1) $$y_{i}=\underset{x_{j}\in \chi \left(i\right)}{\sum }\rho \left(\gamma \left(\varphi \left(x_{i}\right)-\psi \left(x_{j}\right)+\delta \right)\right)⊙\left(\alpha \left(x_{j}\right)+\delta \right)$$

(2) $$\delta =\theta \left(p_{i}-p_{j}\right)$$

where *φ, ψ*, and *α* are linear layers; *γ* and *θ* are nonlinear MLPs with two linear layers and one ReLU layer, respectively; ⊙ is the vector dot product; ẟ is the relative position encoding of two points, denoted as $p_{i}$, $p_{j}$, where $p_{i}$, $p_{j}$ are the three-dimensional coordinates of points *i* and *j*, respectively.

The transition down module reduces the number of points by farthest point sampling [38]. After each transition down module, the number of points becomes one-quarter of the original. We used the transition down module with the same architecture and parameters as the original point transformer. The specific architectures of the transition down module and point transformer module are shown in Figure 2.

### 3.2. Decoder

To generate the point cloud of the occluded part and complete the point cloud, we adopted an improved fold-based decoder architecture as the decoder of the network. The decoder transforms the 3D lattice into a point cloud of the occluded part of the shape. As shown in Figure 1, the global feature of the (1, V) output from the encoder is first repeated M times to form an M × V matrix, which is spliced with the coordinates of the three-dimensional lattice into an M × (V + 3) matrix. The 3D lattice is a cube with coordinates ranging from −1 to 1. There are M points in the cube. Second, the spliced matrix is input into the three-layer perceptron to complete the first deformation. The output 3D coordinates are spliced with the global features and copied M times to obtain an M × (V + 3) matrix, which is input into the three-layer perceptron to achieve the second deformation. Finally, the network outputs the reconstructed point cloud of the occluded part and splices it with the point cloud of the unoccluded part to obtain the completion result.

The decoder part implements a mapping from the 3D lattice to the missing part of the point cloud shape. The global feature output by the encoder serves as a parameter to guide the deformation operation of the decoder, essentially storing the force required to perform deformation. Because the multilayer perceptron is effective at approximating nonlinear functions, it can precisely apply the required force to deform the 3D point cloud and deform the 3D lattice into any desired shape.

### 3.3. Loss Function

The loss function measures the difference between two point clouds. Owing to the disordered nature of point clouds, the loss function should be insensitive to the order of points. We used the Chamfer distance (*CD*) proposed by Fan et al. [34] as our loss function.

(3) $$CD\left(S_{1},S_{2}\right)=\frac{1}{\left|S_{1}\right|}\underset{x\in S_{1}}{\sum }\underset{y\in S_{2}}{\min}∥x-y{∥}_{2}+\frac{1}{\left|S_{2}\right|}\underset{x\in S_{2}}{\sum }\underset{y\in S_{1}}{\min}∥y-x{∥}_{2}$$

Equation (3) is a symmetrical version of the formula used to calculate the *CD* between two point clouds. It measures the average closest point distance between the output point cloud (*S*_1_) and the GT point cloud (*S*_2_). The first term forces the output points to lie close to the GT points and the second term ensures that the GT point cloud is covered by the output point cloud.

In our experiment, we first calculated the *CD* distance between the point cloud *Y_occ_* of the occluded part that is output by the network and the point cloud of the occluded part in the GT (denoted as $GT_{occ}$). This distance is denoted as $Loss_{occ}$. We optimized the network according to $Loss_{occ}$. Then, the output $Y_{occ}$ was spliced with the input point cloud *X* of the unoccluded part to obtain the complete point cloud $Y_{comp}$. The effect of point cloud completion was evaluated by calculating the *CD* between the $Y_{comp}$ and GT.

## 4. Experience and Results

In this section, we first describe how to create a dataset for training our network. We then compare the experimental results of our network with those of FoldingNet. Finally, we describe the ablation experiments used to verify the effectiveness of various changes in our network.

### 4.1. Environment and Network Parameters

#### 4.1.1. System Environment

We implemented our analysis on a PC with Ubuntu18.04 as the operating system, an Intel Core i7-6800K CPU (Intel Technology (China) Co., Ltd.), and an NVIDIA GTX 1080Ti GPU (NVIDIA Semi-Conductor Technology (Shanghai) Co., Ltd.), and the experimental frameworks were python 3.8 (google. Inc.) and pytorch 1.8.2 (Open source software).

#### 4.1.2. Network Specific Parameters

The number of points in the input of the unoccluded part (*N*) was 1536. The number of the transition down and point transformer modules (n) was set to four, and the encoder output a global feature vector with the shape (1512). The cube lattice input to the decoder was set to 512 points with sides of 8 units. Thus, the occluded part of the point cloud output by the decoder contained 512 points. Finally, it was spliced with the unoccluded part to obtain a point cloud of 2048 points.

#### 4.1.3. Model Training Parameters

Adam was used as the network optimizer. The batch size was set to 10; the initial learning rate was set to10^−4^; and after every three rounds of training, the learning rate was reduced to 0.9. The network tended to converge after approximately 200 rounds. To ensure that the network was optimal, we conducted 300 training rounds.

### 4.2. Data Generation and Implementation Detail

To train our network, we used the car categories from the standard ShapeNet dataset. This category has 3162 shapes, and we used 2458 as the training set and 704 as the test set. We uniformly sampled 2048 points of the CAD model of the shape to obtain point cloud data.

**Figure 3:**
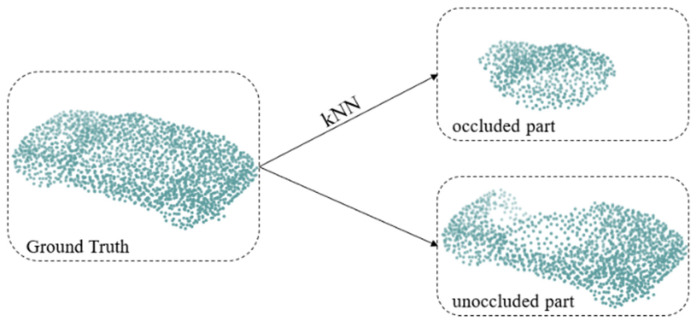
Details of data generation.

All point cloud data were centered at the origin, and the coordinates were normalized to the range [−1, 1]. As shown in Figure 3, we used the sampled 2048 points as the GT of the complete point cloud and deleted 512 points near a random point using the k-nearest neighbor method to simulate occlusion. We used the deleted 512 points as the GT for training the network (denoted as *GT_occ_*), and the remaining 1536 points were used to simulate the unoccluded part as the input of the network.

### 4.3. Results

**Figure 4:**
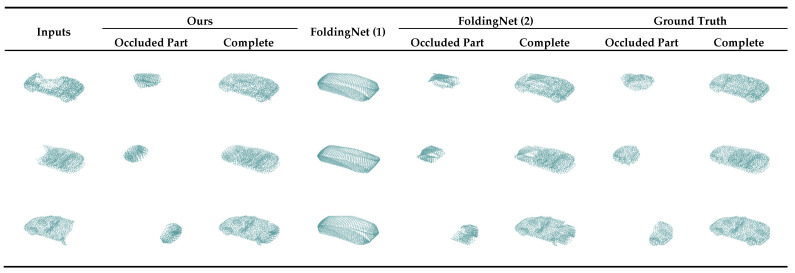
Output results of different methods. Occluded part represents the predicted point cloud of the occluded part, complete represents the complete point cloud after splicing with the unoccluded part. Since FoldingNet (1) was trained using the first method, which directly outputs the completion point cloud, there is no occluded part column.

In this subsection, we qualitatively and quantitatively compare the experimental results of our network with those of the FoldingNet. FoldingNet was trained in two ways: the first was inputting the unoccluded part point cloud and directly predicting the complete point cloud. The 2D lattice of the decoder was initialized with a size of 32 × 64 to output 2048 points. In this experiment, we compared the original completion method of FoldingNet; see the FoldingNet (1) column in Figure 4 and the first row in Table 1. The second method was inputting the unoccluded part of the point cloud and predicting only the occluded part. The two-dimensional lattice in the decoder was initialized with a size of 16 × 32 to output 512 points. This experiment showed that under the same input and output, our completion results were better than those of FoldingNet; see the FoldingNet (2) column in Figure 4 and the second row in Table 1, where $CD_{occ}$ is the *CD* between the predicted occluded point cloud and *GT_occ_*, and $CD_{comp}$ is the *CD* between the complete point cloud and GT.

In Figure 4, we present a visualization of the results of our method and of the two FoldingNet training methods. From this, we can observe that the point cloud density output obtained using our method was more reasonable. The texture and distribution of the point cloud output by our method were closer to the GT and could be combined with the unoccluded parts without being obtrusive. In Table 1, we quantitatively compare the proposed method with FoldingNet and show that the *CD* of our method was much smaller than that of FoldingNet, which directly outputs the complete point cloud. Compared with FoldingNet, which outputs the occluded parts, the *CD* of our method was 8% smaller for the predicted occluded parts, and the *CD* of the complete point cloud output by our method was significantly smaller. These findings indicate that our method outperforms FoldingNet, both visually and quantitatively, suggesting that the improvements we proposed for the original network are effective.

### 4.4. Ablation Study

In this study, we verified the effectiveness of each of our changes through ablation experiments and qualitatively and quantitatively analyzed the experimental results. The datasets quantitatively analyzed the experimental results. The datasets used in the experiments were the point cloud shapes obtained from car models in ShapeNet. We chose the *CD* loss as the evaluation metric.

#### 4.4.1. Transformer Encoder

In this subsection, we evaluate the effectiveness of the transformer encoder in extracting point cloud features. We replaced the transformer encoder with the encoder originally used by FoldingNet and did not change the other parts of the network structure. Comparing the data in the first and second rows in Table 2, after replacing the transformer module, the *CD* loss of the occluded part and complete point cloud increased. This showed that the transformer module has better information extraction ability and improves the performance of the entire network.

#### 4.4.2. 3D Lattice

**Figure 5:**
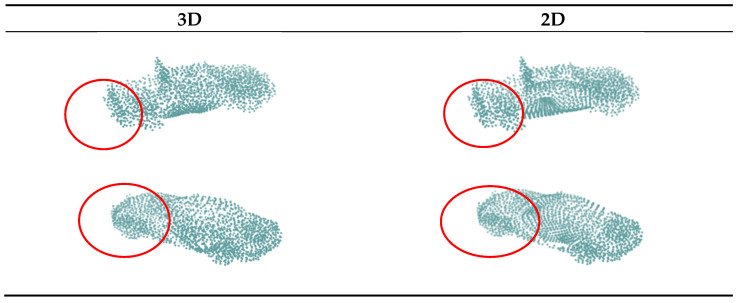
Comparison of using 3D versus 2D lattice.

In this subsection, we evaluate the effectiveness of the 3D lattices in the decoder. We replaced the 3D lattice with the original 2D lattice in the FoldingNet decoder, while the other parts of the network structure remained unchanged. Comparing the data in the first and fourth rows in Table 2, after replacing the 3D lattice with a 2D lattice, the *CD* loss of the occluded part increased by 15.5%. In addition, as shown in the circled part in Figure 5, compared with the 2D lattice, the output point cloud density of the 3D lattice was more uniform, and the connection with the unoccluded part was more natural and unified in vision.

## 5. Discussion

### 5.1. Some Poor Completion

**Figure 6:**
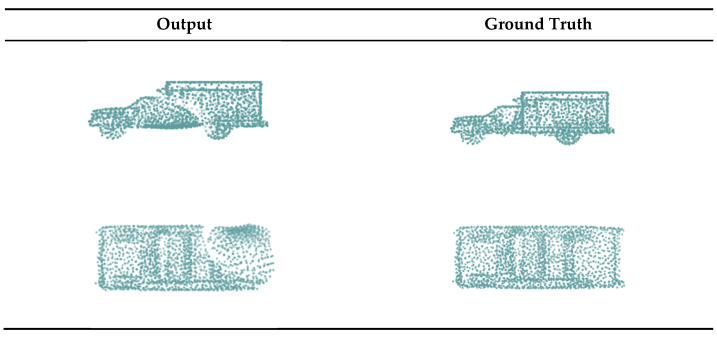
Some point cloud shapes with poor completion.

For most point cloud shapes, the point cloud density of the occluded part output by our network was relatively uniform and could be smoothly spliced with the unoccluded part; however, the distribution of the output point cloud was still slightly different from that of the occluded part. In some shapes, there was a gap between the point cloud of the occluded part and that of the unoccluded part (as shown in Figure 6). We speculate that this is because the network does not fully learn the distribution rules of point clouds in space, resulting in a certain degree of difference in the distribution between the output and original point clouds. If the distribution of the output point cloud of the generative adversarial network is close as possible to the original point cloud, the results may be improved.

### 5.2. The Effect of Density

In theory, the higher the density, the more conducive the network is to extracting the features of the point cloud and the better the completion effect of the point cloud. To verify the effect of density on the performance of the network, we conducted experiments with point clouds with 1024 points, and compared it with the experiments in Section 4 (using point clouds of 2048 points). The data showed that using the point clouds with 1024 points for training, the *CD* of the occluded part was 5.272 × 10^−2^, which is an increase compared with the 4.104 × 10^−2^ in the previous experiment.

**Figure 7:**
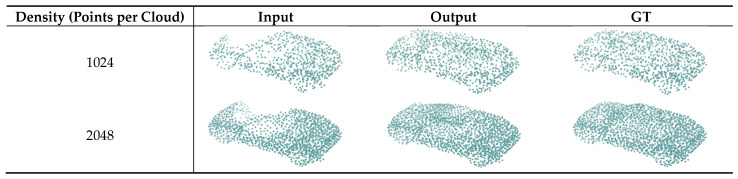
The completion shape of different densities.

In short, high-density point clouds are more conducive to completion. The shape of the completion is shown in Figure 7.

### 5.3. The Effect of the Scale of Occlusion

In theory, the more occluded parts, the more difficult it is to extract the features of the point cloud, and the completion effect may be affected to a certain extent. To verify the effect of the volume of the occluded part on network performance, we designed an experiment with 50% occlusion. The experimental results showed that under 50% occlusion, the *CD* of the missing part was 4.112 × 10^−2^, while the *CD* of the missing part in Section 4 (25% occlusion) was 4.104 × 10^−2^. The results showed that as the degree of occlusion increased, the *CD* only slightly increased, indicating that the network we designed could still effectively extract the global features of objects from severely occluded data.

**Figure 8:**
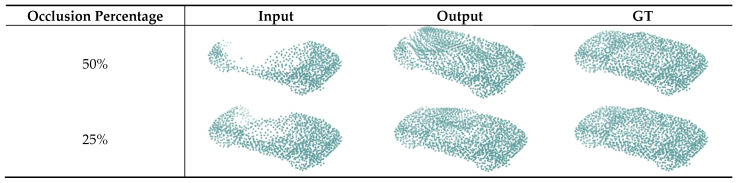
The completion shape of different occlusion percentages.

In short, the larger the volume of the occluded part, the worse the completion performance. The shape of completion is shown in Figure 8.

### 5.4. The Behavior of the Network on the Other Categories

To verify that our network works on other categories of shapes, we conducted experiments on seven other categories, and the results are shown in Table 3.

Among the seven categories, performance in the airplane category was the best, and performance in the cabinet category was the worst. The network produced different performances for the different categories of shapes. We think that for objects with more details, it is more difficult for the network to extract the detailed features, and the shape is more difficult to fit. Overall, our network is usable in other categories as well.

## 6. Conclusions

This study proposed an end-to-end deep neural network for point cloud completion. Our network improved upon FoldingNet. We used a transformer as the encoder of the point cloud completion network to extract the global features of the point cloud; we also replaced the 2D lattice in the decoder with a 3D lattice for the output point cloud density to be more uniform and detailed. We conducted experiments on a point cloud dataset sampled from the ShapeNet car category model. The experimental results showed that the changes we made to the cloud completion network improved its performance. The point cloud completion network proposed in this study can enable machines to acquire and analyze information about surrounding objects with increased accuracy and improve their perception of their surroundings.

## Figures and Tables

**Table 1:** Quantitative comparison of different methods.

Methods	$CD_{occ}\left(\times 10^{-2}\right)\;$	$CD_{comp}\left(\times 10^{-2}\right)$
FoldingNet (1)	—	4.403
FoldingNet (2)	4.461	1.034
Ours	4.104	0.965

**Table 2:** Ablation study.

Methods	$CD_{occ}\left(\times 10^{-2}\right)$	$CD_{comp}\left(\times 10^{-2}\right)$
Ours	4.104	0.964
Without Transformer	4.228	0.984
Without 3D	4.747	1.088

**Table 3:** The behavior of the network on different categories.

Category	$CD_{occ}\left(\times 10^{-2}\right)$
Airplane	2.720
Cabinet	6.099
Chair	5.965
Lamp	5.925
Sofa	5.374
Table	5.563
Watercraft	4.411
Car	4.104

## Data Availability

Not applicable.
